# High-Fat Diets Led to OTU-Level Shifts in Fecal Samples of Healthy Adult Dogs

**DOI:** 10.3389/fmicb.2020.564160

**Published:** 2020-12-08

**Authors:** Logan R. Kilburn, Lucas R. Koester, Stephan Schmitz-Esser, Nick V. L. Serão, Mariana C. Rossoni Serão

**Affiliations:** ^1^Department of Animal Science, Iowa State University, Ames, IA, United States; ^2^Interdepartmental Microbiology Graduate Program, Iowa State University, Ames, IA, United States; ^3^Department of Veterinary Microbiology and Preventive Medicine, Iowa State University, Ames, IA, United States

**Keywords:** dogs, high fat diet, low carbohydrate, health, microbiota

## Abstract

High fat diets have been reported to negatively affect the microbiota in both mice and humans. However, there is a lack of studies in canine models. The variation among the gastrointestinal (GI) tract anatomy/physiology and typical diet compositions of these animal species may lead to vastly different results. Due to the large inclusion rate of dietary fat in pet food, it is critical to understand its effects in a canine model. Therefore, the study objective was to report the effects of high fat, low carbohydrate diets on the fecal microbiota in healthy adult dogs. Eight adult beagles were randomly assigned to one of four dietary treatments within each 15-day period of a replicated 4x4 Latin Square design. Diets contained 32% (T1), 37% (T2), 42% (T3), and 47% (T4) fat. T2, T3, and T4 were created by adding increasing levels of canola oil to T1, a commercially manufactured canned canine diet, which served as the control diet. Fresh fecal samples were collected during the last 5 days of each period for microbial analysis. DNA was extracted from fecal samples and paired-end 16S rRNA gene amplicon sequencing was performed using the Illumina MiSeq platform. When comparing whole microbial communities using PERMANOVA, no significant differences were observed among treatments (*P* = 0.735). Individual OTUs were analyzed using the GLIMMIX procedure of SAS with fixed effects of diet and room, and the random effects of period and animal. Out of the 100 most abundant individual OTUs, 36 showed significant differences in abundance based on treatment (*q* < 0.05). Overall, OTUs assigned to genera related to fat digestion increased while OTUs assigned to genera involved in carbohydrate digestion decreased. In conclusion, the microbial community adapted to dietary intervention without jeopardizing the health of the animals, evaluated by body condition score, fecal characteristics, and blood parameters.

## Introduction

The increased interest in the gastrointestinal (GI) tract microbiota in humans has extended to companion animals. This may be due to the idea that a balanced relationship between the GI microbes and the host animal is critical for host health (Mackie et al., 1999; Hooper et al., 2001). The GI tract microbiota is comprised of thousands of interdependent and/or competing microbial species (Eckburg et al., 2005; Ley et al., 2008; Spor et al., 2011), many of them are still not fully characterized (Hand et al., 2013). The GI tract microbiota can benefit the host in many ways; it can enhance metabolic capabilities, protect against pathogens, develop the immune system, and modulate gastrointestinal development (Backhed et al., 2005; McKenney and Pamer, 2015; Rooks and Garrett, 2016; Wernimont et al., 2020). In addition, GI tract microorganisms contain enzymes that digest fiber and carbohydrates that cannot be digested by the host, producing for example short chain fatty acids (SCFA) (Sunvold et al., 1995), which can be used as an additional energy source for the host. SCFA account for ~10% of human caloric requirement and ~80% of maintenance energy for ruminants (Bergman, 1990). Unlike other species, dogs do not rely heavily on microbial fermentation to meet daily energy requirements, even when fed high fiber diets (Swanson et al., 2010; Hooda et al., 2012; Deng and Swanson, 2015). Even though dogs do not rely on this energy source, a balanced microbiota is nevertheless critical for GI health (Swanson et al., 2010). A disruption, or dysbiosis, of the GI tract microbiota has been associated with disease in both humans and dogs including chronic diarrhea (Bell et al., 2008; Jia et al., 2010) and inflammatory bowel disease (IBD) (Nobaek et al., 2000; Janeczko et al., 2008; Xenoulis et al., 2008; Suchodolski et al., 2010). With disease, there is usually specific shifts in microbial population or a decrease in overall diversity, making disturbances in the GI tract microbiota a possible early warning sign for disease (Deng and Swanson, 2015).

To date, most of the research investigating the dog microbiota has analyzed fecal samples from healthy (free of disease with ideal body condition score) Beagle dogs in controlled laboratory settings (Vanhoutte et al., 2005; Middelbos et al., 2010; Handl et al., 2011; Hang et al., 2012; Beloshapka et al., 2013; Deng and Swanson, 2015; Panasevich et al., 2015; Herstad et al., 2017). These studies have shown that bacteria dominate the canine gut microbiota accounting for ~99% of total sequences with archaea accounting for the remaining 1% (Middelbos et al., 2010; Swanson et al., 2010; Garcia-Mazcorro et al., 2011; Handl et al., 2011; Hand et al., 2013). The predominant phyla found in the GI tract of healthy dogs are *Firmicutes, Bacteroidetes, Proteobacteria, Fusobacteria*, and *Actinobacteria* (Suchodolski et al., 2008; Middelbos et al., 2010; Swanson et al., 2010; Hooda et al., 2012; Deng and Swanson, 2015; Herstad et al., 2017; Li et al., 2017; Schauf et al., 2018). However, bacterial species typically indicated as pathogens such as *Clostridium difficile*, C*lostridium perfringens, Enterococcus* spp., *E. coli*, and *Helicobacter* are often considered part of a dog's healthy microbiota (Jia et al., 2010; Handl et al., 2011; Goldstein et al., 2012). The fecal microbiome of the dog reflects the high concentrations of protein and fat in their diets (Moon et al., 2018). For reference, a typical canned dog food contains 20–32% fat, 28–50% protein, and 18–57% carbohydrate on a dry matter basis (Case et al., 2011).

Compared to other nutrients, dietary fat and its effect on the microbiota have been underestimated due to the argument that little dietary fat reaches the colon where the highest density of bacteria reside (Cândido et al., 2018). However, Gabert et al. (2011) showed that free fatty acids were being excreted in healthy people. Free fatty acids are known to have potent antimicrobial effects even at small doses (Huang et al., 2011; Cândido et al., 2018). Therefore, the small amounts of fat reaching the colon could interact with the resident microbiota. In addition, a higher fat content will require an increased amount of bile acids for digestion, which are also known to have an antimicrobial effect (Stacey and Webb, 1947). Specific bacteria are known to be involved with the digestion and absorption of dietary fat. *Lactobacillus, Bifidobacterium, Enterobacter, Bacteroides*, and *Clostridium* are involved in bile acid metabolism and affect the absorption of dietary fats and lipid-soluble vitamins (Ridlon et al., 2006, Swann et al., 2011). *Faecalibacterium prausnitzii* and *Bifidobacterium* are associated with choline metabolism to modulate lipid metabolism and glucose homeostasis (Martin et al., 2010; Wang et al., 2011).

In recent studies, high fat diets are typically associated with a decrease in overall microbial abundance and diversity with a shift from *Bacteroidetes* to *Firmicutes* (Hildebrandt et al., 2009; Zhang et al., 2012; Murphy et al., 2015). This shift may lead to increased gut permeability, inflammation, and disease (Murphy et al., 2015). These aforementioned studies have been conducted using murine models with a lack of evidence in canine or other large animal models. Due to the high inclusion rate of dietary fat in pet foods and the demand for less processed diets (decreased carbohydrates), it is important to understand the role of dietary fat on the canine microbiota. Additionally, a recent study by Coelho et al. (2018) using metagenome shotgun sequencing has determined the genetic potential of canine gut microbial communities (consisting of 1,247,405 non-redundant genes) to be more similar to human gut microbial communities, compared to swine and murine microbiota. The authors suggest that canine models may be more accurate in estimating the impact of dietary intervention on human microbial communities.

The study objective within this manuscript was to evaluate the effects of feeding adult dogs increasing levels of fat in low carbohydrate diets on the fecal microbiome. The hypothesis of this study was that microbial shifts would occur based on microbial adaptation to dietary intervention, but dogs would maintain health status due to their ability to efficiently digest fat. Results concerning diet digestibility, fecal characteristics, and blood parameters of the dogs used in this study have been previously published (Kilburn et al., 2020).

## Materials and Methods

The protocol for this experiment was reviewed and approved by the Iowa State University Institutional Animal Care and Use Committee (IACUC).

### Animals and Housing

Eight female beagles, 1 year of age with an average baseline body weight of 8.57 ± 0.93 kg (mean ± SD) were enrolled in this study. Dogs were spayed prior to the study to prevent any confounding hormonal effects. To ensure that all dogs were healthy before conducting the trial, complete blood count and chemistry panels were performed. In addition, fecal samples were analyzed for parasite presence. All dogs were housed in pairs at the College of Veterinary Medicine at Iowa State University (Ames, IA, USA) in temperature-controlled rooms (20°C) on a 12:12 h light: dark schedule. During feeding and collection periods, dogs were separated by gate closure.

### Diets and Feeding

Dietary compositions are presented in Table 1. A commercially manufactured canned canine diet (Supplementary Table 1) was used as a control. Canola oil was then added to the control at 2%, 4%, and 6%, as fed, to create the additional treatments. Treatment diets contained 32% (T1), 37% (T2), 42% (T3), and 47% (T4) total dietary fat on a dry matter basis. The control diet (T1) was selected as it was already higher in fat and lower in carbohydrates compared to other commercially manufactured diets. As canola oil was added, the estimated carbohydrate content (nitrogen-free extract) of the diets decreased. Diets contained an estimated 6.61% (T1), 7.52% (T2), 5.84% (T3), and 3.71% (T4) nitrogen-free extract. Of note, the calculated nitrogen-free extract of T2 was greater than expected.

**Table 1:** Analyzed chemical composition of dietary treatments (dry matter basis).

**Item**	**Treatment^a^**			
	**T1**	**T2**	**T3**	**T4**
Dry matter, %	22.15	24.85	24.94	26.74
Moisture, %	77.85	75.15	75.06	73.26
Organic matter, %	88.96	90.74	90.63	91.60
Ash, %	11.05	9.27	9.37	8.41
Crude protein, %	46.88	42.72	40.02	38.19
Fat, %	32.05	37.15	41.86	46.49
Total dietary fiber, %	3.41	3.34	3.27	3.20
Nitrogen-free extract^b^, %	6.61	7.52	5.84	3.71
Gross energy, kcal/kg	6068.01	6361.67	6488.54	6705.12

a *T1: 32% fat; T2: 37% fat; T3: 42% fat; T4: 47% fat*.

b *Nitrogen-Free Extract = calculation of estimated carbohydrate content [100 – (ash + crude protein + fat + total dietary fiber)]*.

Dogs were fed twice daily (0800 and 1700 h) to meet their daily energy requirements. Total daily energy requirements were calculated per treatment for each individual dog based on body weight at the beginning of each period. In other words, as dietary fat (energy) of the diet increased less diet was offered. Therefore, as dietary fat increased total carbohydrate consumed decreased. Weight and body condition score (BCS) were recorded weekly. If needed, feed intake was adjusted during the adaption phase to maintain ideal BCS. Water was provided *ad libitum* throughout the study.

### Experimental Design and Sample Collection

Dogs were randomly assigned to one of four dietary treatments in a replicated 4 × 4 Latin Square design. This design allowed each dog to serve as its own control. Each period included a 10-day diet adaption phase followed by a 5-day total collection phase.

During the collection phase, 2 g of fresh feces (defecated within 15 min) were placed into a cryovial tube and immediately stored in −80°C for microbiota analysis for each dog per treatment.

### Fecal DNA Extraction

Fecal samples were thawed, and DNA was extracted from ~0.25 g of feces using the Qiagen DNeasy Powerlyzer Powersoil kit (Germantown, MD) following the manufacturer's instructions. Mechanical cell lysis was performed using a Fischer Scientific Beadmill 24. DNA concentrations were determined using a spectrophotometer (ND-100; NanoDrop Technologies, Inc., Rockland, DE) prior to sequencing. DNA concentrations for all samples are shown in Supplementary Table 2.

After extraction, DNA was sent to the Iowa State University DNA facility for paired-end, 16S rRNA gene amplicon sequencing (V4) using the Illumina MiSeq platform. Briefly, the genomic DNA from each sample was amplified using the Platinum™ Taq DNA Polymerase (Thermo Fisher Scientific, Waltham, MA) with one replicate per sample using universal 16S rRNA gene bacterial primers [515F (5′ GTGYCAGCMGCCGCGGTAA-3′; Parada et al., 2016) and 806R (5′-GGACTACNVGGGTWTCTAAT-3′; Apprill et al., 2015)] amplifying the variable region V4. All samples underwent PCR with an initial denaturation step at 94°C for 3 min, followed by 45 s of denaturing at 94°C, 20 s of annealing at 50°C, and 90 s of extension at 72°C. This was repeated for 35 total PCR cycles and finished with a 10 min extension at 72°C. DNA was then purified of primers, nucleotides, enzymes, mineral oil, salts, agarose, ethidium bromide, and other impurities using the QIAquick 96 PCR Purification Kit (Qiagen Sciences Inc, Germantown, MD). PCR bar-coded amplicons were mixed at equal molar ratios and used for Illumina MiSeq paired-end sequencing with 250 bp read length and cluster generation with 10% PhiX control DNA on an Illumina MiSeq platform (Illumina Inc., San Diego, CA).

### Sequence Analysis

Sequence analysis was done with Mothur V1.40.5 following the Mothur MiSeq SOP (Kozich et al., 2013). Barcode sequences, primer and low-quality sequences were trimmed using a minimum average quality score of 35, with a sliding window size of 50 bp. Chimeric sequences were removed with the “Chimera.uchime” command. For alignment and taxonomic classification of sequences, the SILVA SSU NR reference database v132 provided by the Mothur website was used. The sequences were then clustered into operational taxonomic units (OTUs) based on 99% 16S rRNA gene similarity (=0.01 distance).

Entire microbial communities of each sample were either rarefied to the lowest sequencing depth per sample (20,900), or non-rarefied prior to assigning Bray-Curtis dissimilarity coefficients to perform statistical comparisons between treatment groups. After dissimilarity coefficients were assigned to each sample, treatment groups were compared using the Adonis (PERMANOVA) command from the *vegan* package in R (Oksanen et al., 2019).

Both rarefied and non-rarefied microbial communities were visualized by plotting (ggplot2 v2_3.1.1 graphing package in R 3.6.0; Wickham, 2009; R Core Team, 2019) principle coordinate analysis (PCoA) generated with the Phyloseq (v1.28.0, McMurdie and Holmes, 2013) and *vegan* (v2.5-5) packages using the shared and taxonomy file generated in Mothur. Bray-Curtis dissimilarity measures were used to generate distances between samples for the PCoA plot, then each sample value was plotted.

Canonical analysis of principle coordinates (CAP) (Anderson and Willis, 2003) was conducted to detect any differences in whole microbial communities based on treatment in relation to animal health parameters collected in Kilburn et al. (2020). Once again, Bray-Curtis dissimilarity measures were used to generate distances between samples, which were then constrained based on model effects (treatment, room, and period) and certain animal health measurements (fat digestibility, fecal dry matter, feed intake (as fed), and red blood cell distribution width) collected in the previous study. Animal health measurements used to constrain the data were selected based on (1) significance detected in Kilburn et al. (2020) and (2) correlations detected between these animal parameters. If variables were correlated, a single variable was selected to represent all correlated variables based on study relevance (i.e., fat digestibility representing gross energy digestibility and dry matter digestibility).

To compare alpha diversity between experimental groups, reads were either rarefied to accommodate the sample with the lowest number of reads (20,900 sequences), or non-rarefied similar to entire microbial community comparisons. Measurements of Chao species richness, Shannon diversity, and Simpson evenness were generated within Phyloseq to compare community characteristics between experimental groups. The means of the treatment group alpha diversity measures were compared with ANOVA assuming equal variance.

To create phylum, class, and genus level comparison bar graphs between treatment groups, all sequences agglomerated (tax_glom command in Phyloseq) based on their taxonomic classification assigned via the classify.seqs command using the Silva reference database in Mothur. These sequences were then adjusted to relative abundance values and plotted.

Phyla and individual OTUs were analyzed using the GLIMMIX procedure of SAS (Version 9.4, SAS Inst., Cary, NC) with fixed effects of diet and room, and the random effects of period and animal. A negative binomial was used to determine the distribution with an offset of log library size. *P*-values were transformed to *q*-values using false discovery rate (FDR) correction (Storey, 2002). Q-values were used to determine significance (*q* < 0.05). Orthogonal contrasts were performed on significant phyla and OTUs to determine linear, quadratic, and/or cubic relationships among treatments.

### Statistical Analysis of Body Weight and Body Condition Score

Body weight and BCS were analyzed using the MIXED procedure of SAS (Version 9.4, SAS Inst., Cary, NC) with fixed effects of diet and room, and the random effects of period and animal. Initial body weight or initial body condition score were used as a covariate for their respective analysis. Differences between diets were determined using least squared means. A probability of *P* < 0.05 was considered statistically significant and standard error of the means (SEM) were determined.

### Data Availability

The 16S rRNA gene sequences have been submitted to the NCBI Sequence Read Archive SRA and are available under the BioProject ID PRJNA630443.

## Results

### Body Weight and Body Condition Score

Body weight and BCS are presented in Table 2. Mean body weight (*P* = 0.199) and BCS (*P* = 0.907) of dogs were maintained throughout treatments.

**Table 2:** Body weight and body condition score of dogs per dietary treatment.

**Item**	**Treatment^a^**				**SEM^b^**	***P*-value**
	**T1**	**T2**	**T3**	**T4**		
Body weight, kg	7.66	7.50	7.53	7.55	0.18	0.199
Body condition score	3.63	3.56	3.56	3.50	0.24	0.907

a *T1: 32% fat; T2: 37% fat; T3: 42% fat; T4: 47% fat*.

b *SEM: standard error of the mean*.

### Fecal Microbial Communities

**Figure 1:**
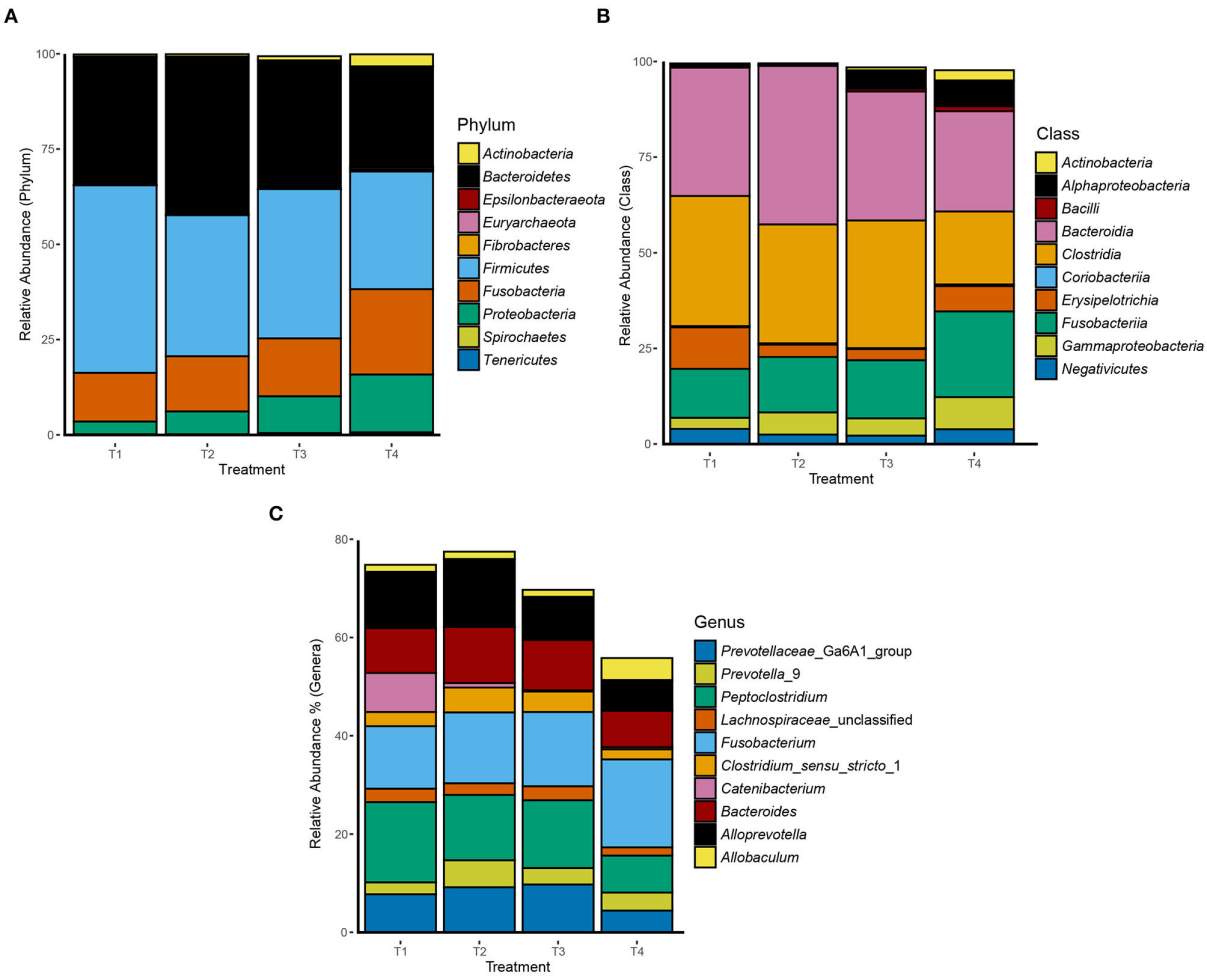
Relative abundance of the 10 most abundant **(A)** phyla, **(B)** classes, and **(C)** genera in dog fecal samples per dietary treatment.

Overall, 2,438 OTUs were generated after quality control and removal of OTUs representing <10 sequences. The average number of sequences per samples was 59,783 with a standard deviation of 25,370. 99.9% of the reads were bacterial while only 0.1% were archaeal. From the 2,438 OTUs, 25 phyla were identified with *Firmicutes* (40%), *Bacteroidetes* (34%), *Fusobacteria* (17%), *Proteobacteria* (7%), and *Actinobacteria* (1%) being the most abundant. The most abundant phyla, classes, and genera per treatment are presented in Figure 1. The classes *Bacteroidia, Clostridia, Fusobacteriia*, and *Erysipelotrichia* accounted for 33%, 30%, 17%, and 6% of total reads, respectively. Additionally, the *Fusobacterium* genus accounted for 18% of all reads. OTU 1 was classified into the *Peptoclostridium* genus which accounted for 14% of total reads. Several OTUs were classified within the genera *Bacteroides* and *Alloprevotella*, with each accounting for 10% of all reads. In addition, the genus *Allobaculum* accounted for 3% of the total reads. The assigned classifications of the 50 most abundant OTUs are presented in Supplementary Table 3.

**Figure 2:**
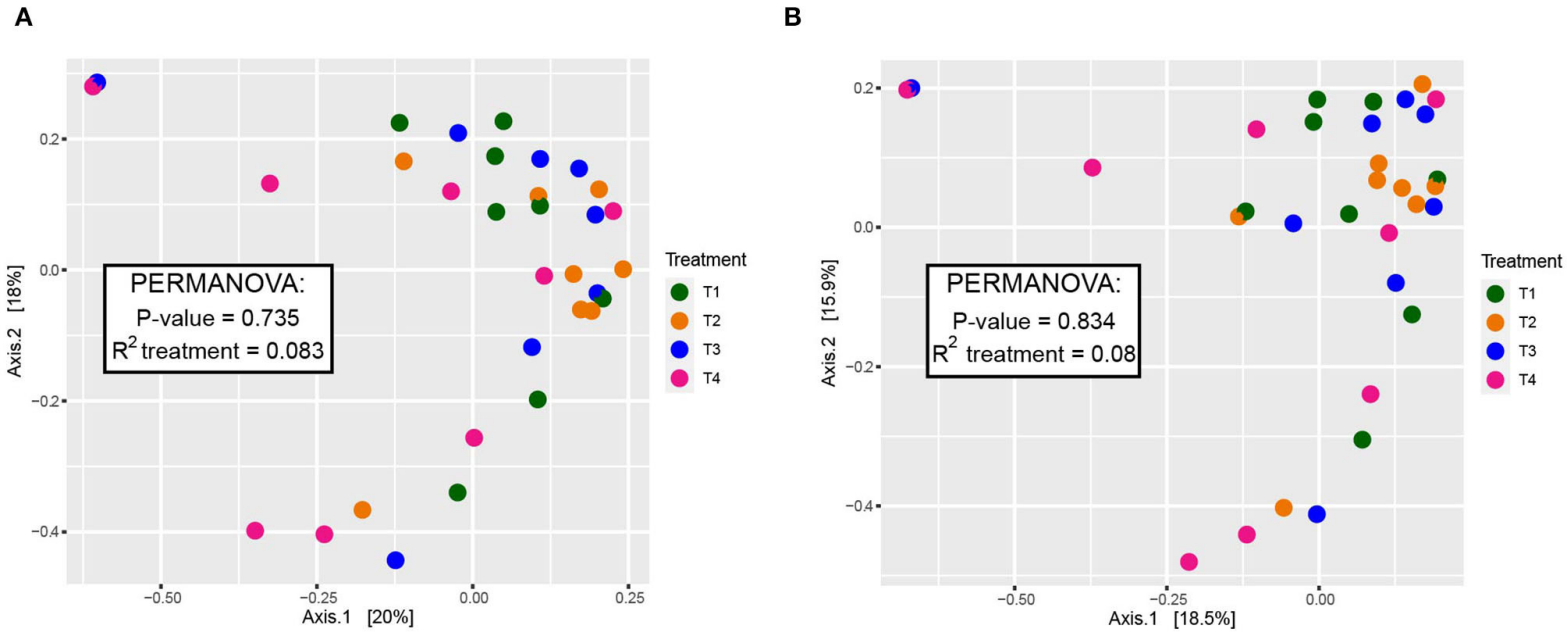
Beta diversity of dog fecal microbial communities revealed by a PCoA plot based on Bray-Curtis dissimilarities of the overall composition of microbial communities among dietary treatment groups. **(A)** Rarefied to 20,900 sequences and **(B)** non-rarefied. Each point corresponds to a community from a single dog. Colors represent each treatment. A summary of PERMANOVA results (*P*-value and *R*^2^) are displayed with the boxes. Additional PERMANOVA information can be found in Supplementary Table 4.

**Figure 3:**
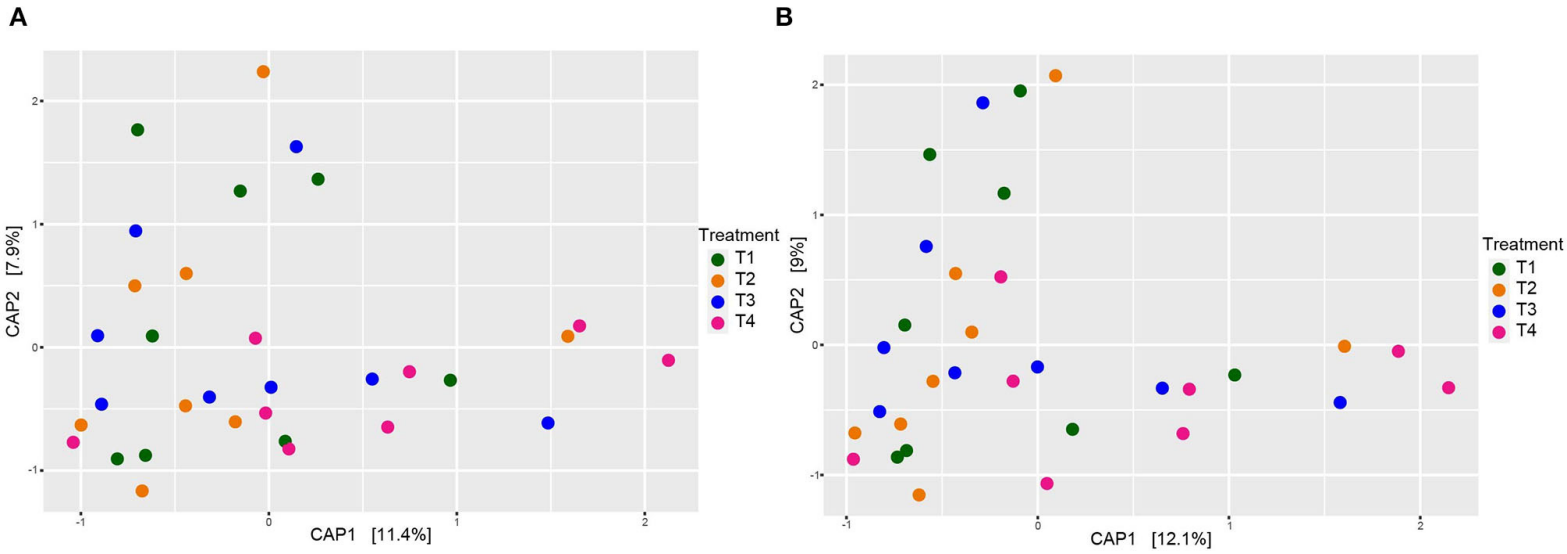
Beta diversity of dog fecal microbial communities revealed by a CAP plot based on Bray-Curtis dissimilarities of the overall composition of microbial communities among dietary treatment groups adjusted by animal health parameters collected by Kilburn et al. (2020). **(A)** Rarefied to 20,900 sequences and **(B)** non-rarefied. Each point corresponds to a community from a single dog. Colors represent each treatment.

When comparing entire bacterial communities of treatment groups using PERMANOVA, no significant differences were observed in either the rarefied or non-rarefied data (*P* = 0.735 and *P* = 0.834, respectively, Supplementary Table 4). This result was supported by the lack of apparent clustering of samples based on treatment types seen in the unconstrained PCoA (Figure 2). Additionally, although we selected animal health measurements that relate to GI microbiota, no clear trends were detected across treatment when constrained by these parameters (Figure 3). Finally, no significant treatment differences were detected across treatment for alpha diversity estimators (Table 3) either.

**Table 3:** Alpha diversity estimators of dog fecal microbial communities-rarefied and non-rarefied.

**Rarefied (20,9000 sequences)**	**Treatment^a^**				**SEM^b^**	***P*-value**
	**T1**	**T2**	**T3**	**T4**		
No. of observed OTUs	779.50	797.50	588.63	633.00	137.79	0.3018
Chao (species richness)	1410.39	1578.38	1088.71	1199.65	243.95	0.2446
Shannon (diversity)	3.47	3.58	3.34	3.33	0.25	0.7819
Simpson (evenness)	0.88	0.91	0.90	0.89	0.03	0.7048
**Non-rarefied**	**Treatment^a^**				**SEM^*b*^**	***P*****-value**
	**T1**	**T2**	**T3**	**T4**		
No. of observed OTUs	1825.38	2017.75	1268.00	1447.13	348.33	0.2379
Chao (species richness)	5442.90	5730.74	3853.06	4465.05	1071.48	0.3833
Shannon (diversity)	3.52	3.62	3.38	3.36	0.25	0.7788
Simpson (evenness)	0.88	0.91	0.90	0.89	0.03	0.7156

a *T1: 32% fat; T2: 37% fat; T3: 42% fat; T4: 47% fat*.

b *SEM: standard error of the mean*.

However, significant differences (*q* < 0.05) in the relative abundance of certain phyla were detected between treatment groups (Table 4). Treatment differences were reported for phyla *Tenericutes* (*q* = 0.0052), *Spirochaetes* (*q* < 0.001), *Euryarchaeota* (*q* = 0.0007), *Fibrobacteres* (*q* < 0.001), *Kiritimatiellaeota* (*q* < 0.001), *Deferribacteres* (*q* < 0.001), and *Planctomycetes* (*q* < 0.001). *Tenericutes, Spirochaetes, Fibrobacteres*, and *Planctomycetes* increased linearly with the increase in dietary fat (*P* < 0.05).

**Table 4:** Relative abundance of significant^a^ phyla among treatments of dog fecal samples.

**Phylum**	**Treatment^b^ relative abundance (%)**				**SEM^*d*^**	***q*-value**	***P-value^c^***		
	**T1**	**T2**	**T3**	**T4**			**Linear**	**Quadratic**	**Cubic**
*Tenericutes*	0.09	0.07	0.42	0.12	0.14	0.0052	0.0229	0.2755	0.0017
*Spirochaetes*	0.05	0.07	0.09	0.53	0.11	<0.0001	<0.0001	0.0152	0.5608
*Euryarchaeota*	0.01	0.01	0.00	0.51	0.11	0.0007	0.8627	0.3716	0.2873
*Fibrobacteres*	0.01	0.00	0.01	0.46	0.11	<0.0001	<0.0001	0.0033	0.8987
*Kiritimatiellaeota*	0.02	0.01	0.00	0.00	0.01	<0.0001	0.0935	0.5495	0.3487
*Deferribacteres*	0.00	0.01	0.00	0.00	0.00	<0.0001	0.9222	0.1849	0.3735
*Planctomycetes*	0.00	0.00	0.01	0.00	0.00	<0.0001	0.0008	<0.0001	0.9178

*Relative abundances of phyla are indicated for each dietary treatment*.

a *q <0.05*.

b *T1: 32% fat; T2: 37% fat; T3: 42% fat; T4: 47% fat*.

c *P-values of orthogonal contrasts*.

d *SEM: standard error of the mean*.

**Figure 4:**
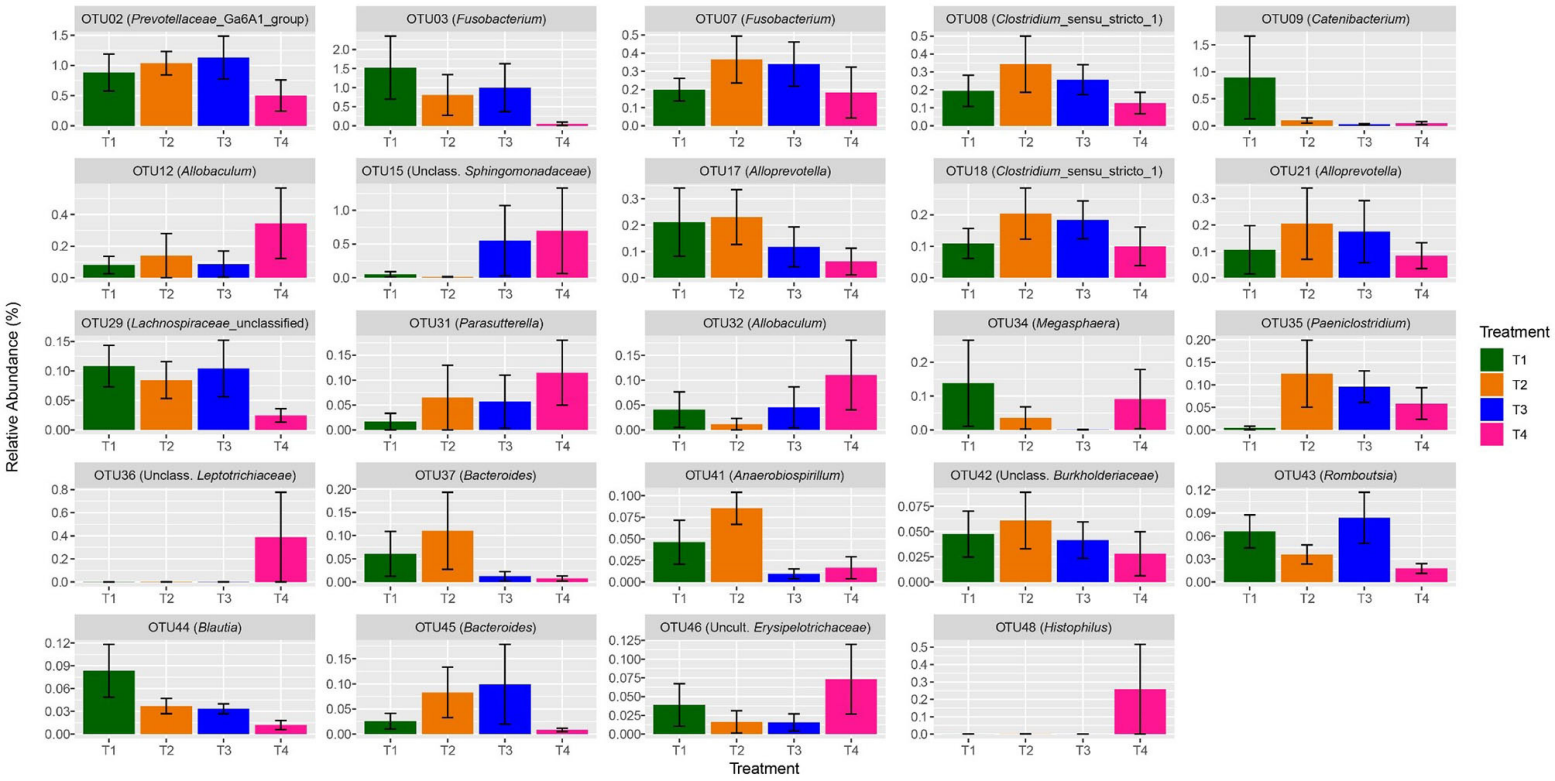
Bar charts presenting a visual representation of the shifts in relative abundance of significant (*q* < 0.05) OTUs per dietary treatment among the 50 most abundant OTUs of dog fecal samples. Error bars represent the standard error among samples (see Table 4 for details).

Similarly, when comparing the abundance of the 100 most abundant individual OTUs between treatment levels, 36 OTUs showed significant (*q* < 0.05) differences (Table 5). For example, treatment differences were reported among the genera *Catenibacterium* (*q* < 0.037), *Paeniclostridium* (*q* = 0.010), *Romboutsia* (*q* = 0.024), *Blautia* (*q* = 0.024), and *Lactobacillus* (*q* < 0.001). A visual representation of significant shifts in OTU abundance per treatment among the 50 most abundant OTUs is presented in Figure 4.

**Table 5:** Relative abundance of significant^a^ OTUs among treatments out of 100 most abundant OTUs of dog fecal samples.

**OTU**	**Genus**	**Treatment^b^ relative abundance (%)**				**SEM^d^**	***q*-value**	***P*****-value^c^**		
		**T1**	**T2**	**T3**	**T4**			**Linear**	**Quadratic**	**Cubic**
2	*Prevotellaceae_Ga6A1_group*	7.06	8.29	9.06	4.00	2.23	0.0195	0.0293	0.0034	0.6046
3	*Fusobacterium*	12.20	6.46	8.00	0.40	4.08	<0.0001	<0.0001	0.9029	0.2161
7	*Fusobacterium*	1.59	2.92	2.72	1.46	0.91	0.0235	0.0689	0.0025	0.7787
8	*Clostridium_sensu_stricto_1*	1.56	2.75	2.06	1.02	0.77	0.0184	0.2242	0.0008	0.7351
9	*Catenibacterium*	7.15	0.78	0.22	0.38	1.72	0.0373	0.0099	0.0274	0.9053
12	*Allobaculum*	0.65	1.12	0.69	2.75	1.00	<0.0001	<0.0001	<0.0001	<0.0001
15	*Sphingomonadaceae_unclassified*	0.42	0.11	4.40	5.57	2.40	0.0004	0.0001	0.5731	0.0005
17	*Alloprevotella*	1.69	1.84	0.94	0.50	0.72	0.0036	0.0168	0.0101	0.0060
18	*Clostridium_sensu_stricto_1*	0.87	1.63	1.47	0.80	0.50	0.0122	0.3925	0.0004	0.8144
21	*Alloprevotella*	0.85	1.64	1.40	0.67	0.79	<0.0001	<0.0001	0.0004	0.0364
29	*Lachnospiraceae_unclassified*	0.87	0.68	0.83	0.20	0.25	0.0047	0.0008	0.0312	0.0666
31	*Parasutterella*	0.13	0.52	0.45	0.92	0.40	<0.0001	<0.0001	0.0022	0.0028
32	*Allobaculum*	0.33	0.09	0.36	0.88	0.32	<0.0001	<0.0001	<0.0001	0.6165
34	*Megasphaera*	1.10	0.28	0.01	0.73	0.50	0.0005	0.1001	0.0019	0.0001
35	*Paeniclostridium*	0.03	1.00	0.77	0.47	0.30	0.0095	0.0048	0.0065	0.3056
36	*Leptotrichiaceae_unclassified*	0.00	0.01	0.01	3.11	0.78	<0.0001	<0.0001	0.0043	0.0174
37	*Bacteroides*	0.48	0.88	0.10	0.06	0.29	0.0184	0.0020	0.1615	0.0615
41	*Anaerobiospirillum*	0.37	0.68	0.08	0.13	0.12	0.0128	0.0042	0.9391	0.0118
42	*Burkholderiaceae_unclassified*	0.38	0.49	0.33	0.22	0.18	0.0282	0.0049	0.0375	0.7818
43	*Romboutsia*	0.53	0.29	0.67	0.14	0.15	0.0242	0.0106	0.1606	0.0254
44	*Blautia*	0.67	0.30	0.27	0.10	0.11	0.0241	0.0011	0.5788	0.2661
45	*Bacteroides*	0.21	0.66	0.79	0.07	0.30	0.0002	0.0058	<0.0001	0.5725
46	*Uncultured_Erysipelotrichaceae*	0.31	0.13	0.12	0.59	0.20	0.0028	0.0232	0.0002	0.8640
48	*Histophilus*	0.01	0.01	0.00	2.07	0.52	0.0014	0.0006	0.0024	0.0156
57	*Erysipelotrichaceae_unclassified*	0.10	0.00	0.18	0.49	0.14	<0.0001	<0.0001	<0.0001	<0.0001
58	*Allobaculum*	0.34	0.21	0.23	0.06	0.09	0.0007	0.0001	0.0057	0.0577
60	*Allobaculum*	0.06	0.00	0.08	0.46	0.10	<0.0001	0.1017	0.2163	0.3553
62	*Succinivibrionaceae_UCG-001*	0.01	0.01	0.87	0.49	0.27	0.0002	<0.0001	0.2991	0.0080
66	*Lachnoclostridium*	0.30	0.15	0.14	0.10	0.09	0.0369	0.0012	0.8364	0.6721
69	*Prevotella_9*	0.14	0.12	0.00	0.66	0.17	0.0377	0.7487	0.0228	0.0102
72	*Leptotrichiaceae_unclassified*	0.00	0.00	0.00	0.94	0.23	<0.0001	<0.0001	0.0006	0.0491
86	*Lachnospiraceae_ge*	0.13	0.27	0.03	0.02	0.09	0.0019	<0.0001	0.2238	0.1004
90	*Bifidobacterium*	0.05	0.01	0.07	0.14	0.05	<0.0001	0.0002	<0.0001	0.0509
96	*Alloprevotella*	0.10	0.09	0.05	0.02	0.03	0.0431	0.0045	0.1189	0.5410
97	*Clostridium Family_XIII_unclassified*	0.07	0.07	0.08	0.03	0.05	0.0050	0.1264	0.0009	0.0094
99	*Lactobacillus*	0.00	0.00	0.16	0.34	0.12	0.0002	<0.0001	0.7223	0.0074

*Relative abundances of OTUs are indicated for each dietary treatment*.

a *q <0.05*.

b *T1: 32% fat; T2: 37% fat; T3: 42% fat; T4: 47% fat*.

c *P-values of orthogonal contrasts*.

d *SEM: standard error of the mean*.

OTUs 12, 15, 31, 32, 35, 36, 46, 48, 57, 62, 72, 90, and 99 significantly (*P* < 0.05) increased in abundance from T1 to T4 (Table 5). OTUs 12 and 32 (*P* < 0.001) were assigned to the genus *Allobaculum*, of the *Firmicutes* phylum. Within the *Proteobacteria* phylum, OTU 15 (*P* < 0.001), OTU 31 (*P* < 0.001), OTU 48 (*P* = 0.001), and OTU 62 (*P* < 0.001) were assigned to *Sphingomonadaceae unclassified, Parasutterela, Histophilus*, and *Succinivibrionaceae_UCG-001*, respectively. OTU 35 (*P* = 0.005) was assigned to the *Paeniclostridium* genus of the *Firmicutes* phylum.

Other OTUs showed a significant (*P* < 0.05) linear decrease in abundance with increasing fat content in the diet, these included OTUs 2, 3, 9, 17, 21, 29, 37, 41, 42, 43, 44, 45, 58, 66, 86, and 96 (Table 5). OTU 2 was assigned to *Prevotellaceae Ga6A1 group* (*P* = 0.029). OTU 3 was assigned to the genus *Fusobacterium* (*P* < 0.001) of the *Fusobacteria* phylum. OTU 9 was assigned to *Catenibacterium* (*P* = 0.010). OTUs 17, 21, and 96 were assigned to the genus *Alloprevotella* (*P* = 0.017, *P* < 0.001, *P* = 0.005, respectively). OTU 29 (*P* = 0.001) and OTU 86 (*P* < 0.001) were assigned to the *Lachnospiraceae* family. OTU 37 and OTU 45 were both assigned to the genus *Bacteroides* (*P* = 0.002, *P* = 0.006). Most of the genera which significantly decreased in abundance belong to the *Bacteroidetes* or *Firmicutes* phylum. OTUs 2, 17, 21, 37, 45, and 96 were all classified to genera within the *Bacteroidetes* phylum while OTUs 9, 29, 43, 44, 58, 66, and 86 all belong to the *Firmicutes* phylum.

## Discussion

### Body Weight and Body Condition Score

It is important that dogs maintained ideal body weight and BCS due to known changes in the microbiota with obesity which may confound diet effects (de La Serre et al., 2010; Cândido et al., 2018). In addition, high fat diets and obesity have been shown to have similar effects on the microbiota making it difficult to determine which caused the microbiota to change if modeled together (Ley et al., 2005; Turnbaugh et al., 2006, 2008; Cândido et al., 2018). The maintenance of ideal BCS allowed this study to measure high fat diets independently of obesity.

### Fecal Microbial Communities

We chose to study fecal microbiota as a proxy for gut microbiota as fecal samples make non-invasive periodic measurements possible. One major limitation of using fecal samples is the fact that fecal samples are more representative of the digesta in the lower gut and might not adequately represent important host-microbial interactions specific to different regions of the GI tract (Leite et al., 2020). In addition, 16S rRNA gene amplicon datasets offer only limited information about functional roles of members of these microbial communities. In this manuscript, any connections between the microbial community and a host phenotype were made using previously published work regarding assigned taxonomy.

Overall, on a whole community level, the high-fat diet did not result in significant shifts in the fecal microbial communities regarding alpha and beta diversity. Shannon diversity indices in this study were greater than those previously reported by Handl et al. (2013) but lower than those reported by Schauf et al. (2018) in dogs. Similar to the study reported here, Schauf et al. (2018) found no difference in bacterial species richness or Shannon diversity comparing a low-fat, high-starch diet to a high-fat, low-starch diet in dogs. Additionally, Coelho et al. (2018) observed a more stable microbiota in non-obese dogs similar to the animals used in this experiment. Other studies using mice have reported a decrease in GI tract microbial abundance when fed high fat diets (Hildebrandt et al., 2009; Zhang et al., 2012). Martinez et al. (2009) reported a decrease in species richness in hamsters fed grain sorghum lipid extract. The decrease in species richness may be caused by the antimicrobial effect of fatty acids and/or bile acids (Stacey and Webb, 1947; Huang et al., 2011; Cândido et al., 2018). To summarize, studies that have fed high fat diets to mice have indicated a decrease in microbial richness while those in dogs have reported no difference with increased dietary fat levels. The varying results in species richness may be due to the differences in physiology and typical diet composition of the animal species used in the studies.

Previous studies have shown a range of abundance for the dominant phyla in healthy dogs with 14–48% *Firmicutes*, 12–38% *Bacteroidetes*, 7–44% *Fusobacteria*, 5–23% *Proteobacteria*, and 0.8–1.4% *Actinobacteria* (Suchodolski et al., 2008; Middelbos et al., 2010; Swanson et al., 2010; Herstad et al., 2017; Li et al., 2017; Coelho et al., 2018; Salas-Mani et al., 2018). The total abundance of each phylum found in this study fall within those ranges indicating normal values. A common metric used in obesity studies from humans, mice, and canines is to report significant differences detected in *Bacteroidetes* and *Firmicutes*. Coelho et al. (2018) reported an increase in the *Firmicutes:Bacteroides* ratio with the switch to a diet with increased protein levels and reduced carbohydrate levels. In addition, high fat diets have been reported to decrease *Bacteroidetes* and increase *Firmicutes* in mice (Hildebrandt et al., 2009; Zhang et al., 2012; Murphy et al., 2015) possibly leading to dysbiosis. No differences in *Firmicutes* abundance or *Bacteroidetes* abundance were seen with the shift to the higher fat, lower carbohydrate diet (T4) in this study (Table 4).

The following section of the manuscript will discuss potential contributions of specific OTUs that demonstrated a linear trend (either increasing or decreasing) with treatment. The data reported in this manuscript is gathered from additional peer-review publications, and a summary of the points discussed can be found in Supplementary Table 5.

#### Bacterial Populations That Increased With Increasing Levels of Dietary Fat may Utilize Fat or Bile as Key Metabolic Substrate

OTUs classified as *Allobaculum* showed differing trends with treatment. The abundance of OTUs 12 and 32 increased with increasing fat levels, whereas the abundance of OTU 58 decreased. Using a 99% OTU clustering threshold, OTUs 12, 32, and 58 could be considered different species which would explain the differences in behavior. In other words, even though these OTUs are classified within the *Allobaculum* genus, they may contain different genetic potential, and thus different metabolic capabilities. *Allobaculum* has been suggested to have beneficial effects and contribute to mucus formation (Everard et al., 2014). The presence of *Allobaculum* and its effect on the host differ among studies. Martinez et al. (2009) reported an increase in *Allobaculum* in hamsters fed grain sorghum lipid extract. In contrast, Everard et al. (2014) and Ravussin et al. (2012) found an increase in *Allobaculum* in lower fat diets compared to high fat diets. Jakobsson et al. (2015) questioned the beneficial role of *Allobaculum* by showing that mice had increased mucus permeability with increased abundance. Different physiological properties of closely related phylotypes have been revealed in previous studies (Berry et al., 2012) and highlight the importance of OTU-level analyses of microbiome data.

Four significant OTUs (15, 31, 48, and 62) were classified into genera within the *Proteobacteria* phylum. Previous studies have reported an increase in *Proteobacteria* with increased consumption of a more natural diet (higher in fat) compared to kibble diets in dogs (Herstad et al., 2017; Sandri et al., 2017). In addition, *Parasutterella* (OTU 31) has a potential role in bile acid maintenance and cholesterol metabolism (Ju et al., 2019). The increase in *Parasutterella* may be explained by the higher fat content which will require an increased amount of bile acid secretion for digestion (Di Ciaula et al., 2017).

OTUs 46 and 57 were classified as uncultured or unclassified *Erysipelotrichaceae*. Another study reported a decreased abundance of unclassified members of the *Erysipelotrichaceae* family in hamsters consuming diets containing grain sorghum lipid extract (Martinez et al., 2009). The differing results may be due to the difference in animal species consuming such diets as the GI tract of a hamster is unlike that of a dog.

*Bifidobacterium* (OTU 90) increased in abundance with increased dietary fat level and has been described as beneficial due to its ability to reduce intestinal endotoxins and improve mucosal barrier function (Griffiths et al., 2004; Wang et al., 2006). In addition, the genus *Bifidobacterium* has bile salt hydrolase (BSH) activity (Ridlon et al., 2006), which might be important when growing on high-fat diets. Martinez et al. (2009) reported an increase in *Bifidobacterium* in hamsters fed grain sorghum lipid extract. However, a study feeding mice a high fat diet found a decrease in bifidobacteria (Cani et al., 2007).

The genus *Lactobacillus* (OTU 99) is known to metabolize carbohydrates resulting in lactic acid production (Walter, 2008). The increase in *Lactobacillus* with increase in dietary fat is interesting due its known role in carbohydrate metabolism (Walter, 2008). Related to the study reported in this manuscript, Salas-Mani et al. (2018) and Coelho et al. (2018) reported a decrease in canine GI tract *Lactobacillus* abundance when fat levels were decreased in canine diets. *Lactobacillus* has been reported to have BSH activity (Ridlon et al., 2006). Therefore, the increase in this genus may be due to the increased need for BSH activity for fat digestion and not its proposed role in carbohydrate metabolism. In addition, an increase in *Lactobacillus* is considered beneficial as some *Lactobacillus* species are commonly used as probiotics due to their health-promoting properties. For example, this genus is thought to modulate the immune system and protect the epithelial barrier in the gut (Lebeer et al., 2008).

#### Bacterial Populations That Decreased With Increasing Levels of Dietary Fat may Prefer Other Nutrients as Key Metabolic Substrate

*Fusobacterium* (OTU 3) is reported to utilize amino acids and produce butyrate (Barcenilla et al., 2000; Butowski et al., 2019). In previous studies, *Fusobacterium* has been linked to high protein diets in dogs (Beloshapka et al., 2013; Bermingham et al., 2017) as well as a general high level of presence within canine fecal microbial communities (Coelho et al., 2018; Wernimont et al., 2020).

*Catenibacterium* (OTU 9), *Anaerobiospirillum* (OTU 41), *Romboutsia* (OTU 43), and *Blautia* (OTU 44) can utilize many types of carbohydrates to produce succinic acid, acetic acid, lactic acid, butyric acid, iso-butyric acid, and ethanol depending on the genera (Davis et al., 1976; Kageyama and Benno, 2000; Liu et al., 2008; Gerritsen et al., 2014). In addition, *Alloprevotella* (OTU 17, OTU 21, and OTU 96) is reported to be a saccharolytic bacteria (Qu et al., 2017). A trend was discovered by Salas-Mani et al. (2018), who reported a decrease in *Blautia* species with the switch from a high fat and high carbohydrate to a low fat and high protein diet in canines, but this may have been driven by the decrease in carbohydrates as opposed to fat levels. Pilla and Suchodolski (2020) point out that a decrease in *Blautia* species may be associated with both acute diarrhea and canine IBD. Neither of these symptoms were detected in this trial, however. Yan et al. (2013) suggested an increase in *Catenibacterium* led to increased SCFA production. In this study, the decreased *Catenibacterium* did not affect short chain fatty acid production among treatments (Kilburn et al., 2020).

The genus *Bacteroides* (OTU 37 and OTU 45) is involved in the fermentation of indigestible carbohydrates (Handl et al., 2013), which were minimal in dietary treatments in this study. *Bacteroides* has been reported to increase in humans consuming a Western diet, which is high in fat and sugar (De Filippo et al., 2010; David et al., 2014). Additionally, the *Bacteroides* genus has been reported by Wu et al. (2011), Coelho et al. (2018), and Salas-Mani et al. (2018) to be positively associated with higher levels of dietary protein. The decrease in OTUs 37 and 45 in response to the increase in dietary fat within this study may indicate that *Bacteroides* do indeed rely mainly on protein-rich substrates.

*Lachnoclostridium* has been reported to produce secondary bile acids through bile acid dihydroxylation activity (Ridlon et al., 2015). In the current study, the decrease in this genus is interesting due to the thought that an increase in dietary fat would require greater production of bile acids for digestion. Consequently, an increase in this genus would rather be expected.

#### Potential Use of Canine Models for Human Disease and Nutrition

As noted in the introduction, studying the dog microbiota and its shifts due to dietary intervention or disease is becoming increasingly relevant for understanding both pet and human health. It is only recently that studies have reported more so on canine microbial communities and their genetic potential rather than that of mice and humans. Additionally, Coelho et al. (2018) reported a high similarity between the genetic catalog of canine microbial communities to that of human microbial communities (63% genes mapped from the dog microbial communities to human gene catalog, as opposed to 32.9% for swine and 19.9% for murine microbial communities). More generally, the high similarity of the dog and human GI tract microbiota suggests possible benefits from using canines as models for human disease or dietary intervention.

## Conclusion

In conclusion, the increase in dietary fat and subsequent decrease in carbohydrate levels did not impact the overall microbial diversity in dogs fed dietary treatment. However, the microbiota did shift based on available diet substrate. Even with this microbial shift, dogs remained healthy during the time of the study. This may indicate that the dog microbiota can adapt to high fat diets without creating a dysbiosis. Further research is needed to analyze the functional characteristics of these changes in microbial communities from dogs fed similar dietary treatments.

## Data Availability Statement

The datasets presented in this study can be found in online repositories. The names of the repository/repositories and accession number(s) can be found at: https://www.ncbi.nlm.nih.gov/, PRJNA630443.

## Ethics Statement

The animal study was reviewed and approved by Iowa State University IACUC.

## Author Contributions

MR and LKi conceived and designed the study. LKi conducted the animal trial and sampling. LKo prepared the samples and analyzed the sequence data. LKo and SS-E assisted in interpretation of results. NS contributed to statistical analysis. All authors have reviewed and approved the final version of the manuscript.

## Conflict of Interest

The authors declare that the research was conducted in the absence of any commercial or financial relationships that could be construed as a potential conflict of interest.
