# Supplementary material for: High-Fat Diets Led to OTU-Level Shifts in Fecal Samples of Healthy Adult Dogs
Source: Front Microbiol. 2020 Dec 8;11:564160. doi: 10.3389/fmicb.2020.564160 (PMC7752866; doi:10.3389/fmicb.2020.564160)
Supplement: Supplementary file 1 [file Table_1.DOCX]

**Supplementary Table S1:** Ingredient composition of control dietary treatment

| Treatment | Ingredients |
| --- | --- |
| T1 (Control) | Chicken, chicken broth, chicken liver, carrots, peas, dried egg product, guar gum, carrageenan, ground flaxseed, potassium chloride, salt, cassia gum, minerals (zinc amino acid chelate, iron amino acid chelate, copper amino acid chelate, manganese amino acid chelate, sodium selenite, potassium iodine), vitamins (vitamin E supplement, thiamine mononitrate, niacin supplement, d-calcium pantothenate, vitamin A supplement, riboflavin supplement, biotin, vitamin B12 supplement, pyridoxine hydrochloride, vitamin D3 supplement, folic acid), choline chloride |
