# Supplementary material for: High-Fat Diets Led to OTU-Level Shifts in Fecal Samples of Healthy Adult Dogs
Source: Front Microbiol. 2020 Dec 8;11:564160. doi: 10.3389/fmicb.2020.564160 (PMC7752866; doi:10.3389/fmicb.2020.564160)
Supplement: Supplementary file 2 [file Table_2.DOCX]

| **Supplementary Table S2:** Sample weight used for DNA extraction yields and total number of sequences before and after quality control | | | | |
| --- | --- | --- | --- | --- |
| Sample ID | Extraction Weight | DNA yield  Concentration (ng/µl) | Number of sequences (pre-quality control) | Number of sequences (post-quality control) |
| Dog 1, T1 | 0.25 | 13.6 | 120903 | 60330 |
| Dog 2, T1 | 0.26 | 11.1 | 119937 | 49308 |
| Dog 3, T1 | 0.25 | 44.6 | 177123 | 79742 |
| Dog 4, T1 | 0.24 | 9.5 | 284860 | 121677 |
| Dog 5, T1 | 0.23 | 6.6 | 152066 | 65568 |
| Dog 6, T1 | 0.25 | 12.4 | 268211 | 113599 |
| Dog 7, T1 | 0.23 | 8.1 | 56594 | 26187 |
| Dog 8, T1 | 0.24 | 7.2 | 51444 | 20928 |
| Dog 1, T2 | 0.23 | 15.2 | 72211 | 36375 |
| Dog 2, T2 | 0.25 | 6.2 | 99029 | 43243 |
| Dog 3, T2 | 0.24 | 6.4 | 149869 | 61837 |
| Dog 4, T2 | 0.26 | 18.6 | 242943 | 107713 |
| Dog 5, T2 | 0.23 | 4.5 | 128231 | 51412 |
| Dog 6, T2 | 0.26 | 21.8 | 90050 | 51802 |
| Dog 7, T2 | 0.24 | 10.4 | 75634 | 31115 |
| Dog 8, T2 | 0.26 | 23.3 | 106204 | 50391 |
| Dog 1, T3 | 0.26 | 7.0 | 168151 | 66755 |
| Dog 2, T3 | 0.24 | 30.0 | 320345 | 121433 |
| Dog 3, T3 | 0.24 | 11.0 | 143571 | 54940 |
| Dog 4, T3 | 0.27 | 13.4 | 69692 | 30595 |
| Dog 5, T3 | 0.25 | 6.4 | 72355 | 31887 |
| Dog 6, T3 | 0.24 | 17.4 | 189705 | 66695 |
| Dog 7, T3 | 0.24 | 6.3 | 142729 | 58831 |
| Dog 8, T3 | 0.26 | 6.3 | 147498 | 61267 |
| Dog 1, T4 | 0.24 | 8.5 | 171701 | 68887 |
| Dog 2, T4 | 0.24 | 7.1 | 132527 | 52285 |
| Dog 3, T4 | 0.26 | 12.3 | 156965 | 57312 |
| Dog 4, T4 | 0.24 | 11.7 | 184389 | 73918 |
| Dog 5, T4 | 0.24 | 15.7 | 187666 | 68851 |
| Dog 6, T4 | 0.25 | 8.2 | 208093 | 77379 |
| Dog 7, T4 | 0.26 | 12.1 | 163040 | 60412 |
| Dog 8, T4 | 0.24 | 10.4 | 76957 | 36292 |
