# Supplementary material for: High-Fat Diets Led to OTU-Level Shifts in Fecal Samples of Healthy Adult Dogs
Source: Front Microbiol. 2020 Dec 8;11:564160. doi: 10.3389/fmicb.2020.564160 (PMC7752866; doi:10.3389/fmicb.2020.564160)
Supplement: Supplementary file 3 [file Table_3.DOCX]

| **Supplementary Table S3:** Taxonomic classifications of the 50 most abundant OTUs among dog fecal samples across all samples | | | | |
| --- | --- | --- | --- | --- |
| OTU | Phylum | Family | Genus | Relative Abundance, % |
| 1 | *Firmicutes* | *Peptostreptococcaceae* | *Peptoclostridium* | 13.68 |
| 2 | *Bacteroidetes* | *Prevotellaceae* | *Prevotellaceae_Ga6A1_group* | 7.37 |
| 3 | *Fusobacteria* | *Fusobacteriaceae* | *Fusobacterium* | 6.99 |
| 4 | *Bacteroidetes* | *Prevotellaceae* | *Alloprevotella* | 6.70 |
| 5 | *Fusobacteria* | *Fusobacteriaceae* | *Fusobacterium* | 4.55 |
| 6 | *Bacteroidetes* | *Bacteroidaceae* | *Bacteroides* | 2.77 |
| 7 | *Fusobacteria* | *Fusobacteriaceae* | *Fusobacterium* | 2.24 |
| 8 | *Firmicutes* | *Clostridiaceae_1* | *Clostridium_sensu_stricto_1* | 2.15 |
| 9 | *Firmicutes* | *Erysipelotrichaceae* | *Catenibacterium* | 1.87 |
| 10 | *Fusobacteria* | *Fusobacteriaceae* | *Fusobacterium* | 1.69 |
| 11 | *Bacteroidetes* | *Prevotellaceae* | *Prevotella_9* | 1.68 |
| 12 | *Firmicutes* | *Erysipelotrichaceae* | *Allobaculum* | 1.64 |
| 13 | *Bacteroidetes* | *Prevotellaceae* | *Prevotella_9* | 1.57 |
| 14 | *Firmicutes* | *Lachnospiraceae* | *Lachnospiraceae_ge* | 1.52 |
| 15 | *Proteobacteria* | *Sphingomonadaceae* | *Sphingomonadaceae_unclassified* | 1.49 |
| 16 | *Firmicutes* | *Peptostreptococcaceae* | *Romboutsia* | 1.37 |
| 17 | *Bacteroidetes* | *Prevotellaceae* | *Alloprevotella* | 1.27 |
| 18 | *Firmicutes* | *Clostridiaceae_1* | *Clostridium_sensu_stricto_1* | 1.23 |
| 19 | *Proteobacteria* | *Burkholderiaceae* | *Sutterella* | 1.23 |
| 20 | *Proteobacteria* | *Enterobacteriaceae* | *Escherichia-Shigella* | 1.13 |
| 21 | *Bacteroidetes* | *Prevotellaceae* | *Alloprevotella* | 1.13 |
| 22 | *Firmicutes* | *Veillonellaceae* | *Megamonas* | 1.03 |
| 23 | *Bacteroidetes* | *Bacteroidaceae* | *Bacteroides* | 0.98 |
| 24 | *Bacteroidetes* | *Bacteroidaceae* | *Bacteroides* | 0.94 |
| 25 | *Bacteroidetes* | *Bacteroidaceae* | *Bacteroides* | 0.89 |
| 26 | *Bacteroidetes* | *Bacteroidaceae* | *Bacteroides* | 0.86 |
| 27 | *Firmicutes* | *Acidaminococcaceae* | *Phascolarctobacterium* | 0.85 |
| 28 | *Firmicutes* | *Family_XIII* | *Family_XIII_ge* | 0.69 |
| 29 | *Firmicutes* | *Lachnospiraceae* | *Lachnospiraceae_unclassified* | 0.67 |
| 30 | *Bacteroidetes* | *Bacteroidaceae* | *Bacteroides* | 0.63 |
| 31 | *Proteobacteria* | *Burkholderiaceae* | *Parasutterella* | 0.61 |
| 32 | *Firmicutes* | *Erysipelotrichaceae* | *Allobaculum* | 0.54 |
| 33 | *Firmicutes* | *Peptococcaceae* | *Peptococcus* | 0.49 |
| 34 | *Firmicutes* | *Veillonellaceae* | *Megasphaera* | 0.48 |
| 35 | *Firmicutes* | *Peptostreptococcaceae* | *Paeniclostridium* | 0.48 |
| 36 | *Fusobacteria* | *Leptotrichiaceae* | *Leptotrichiaceae_unclassified* | 0.47 |
| 37 | *Bacteroidetes* | *Bacteroidaceae* | *Bacteroides* | 0.44 |
| 38 | *Firmicutes* | *Lachnospiraceae* | *Blautia* | 0.44 |
| 39 | *Firmicutes* | *Peptostreptococcaceae* | *Peptostreptococcaceae_unclassified* | 0.42 |
| 40 | *Bacteroidetes* | *Bacteroidaceae* | *Bacteroides* | 0.41 |
| 41 | *Proteobacteria* | *Succinivibrionaceae* | *Anaerobiospirillum* | 0.40 |
| 42 | *Proteobacteria* | *Burkholderiaceae* | *Burkholderiaceae_unclassified* | 0.38 |
| 43 | *Firmicutes* | *Peptostreptococcaceae* | *Romboutsia* | 0.38 |
| 44 | *Firmicutes* | *Lachnospiraceae* | *Blautia* | 0.37 |
| 45 | *Bacteroidetes* | *Bacteroidaceae* | *Bacteroides* | 0.35 |
| 46 | *Firmicutes* | *Erysipelotrichaceae* | *uncultured* | 0.35 |
| 47 | *Firmicutes* | *Clostridiaceae_1* | *Clostridium_sensu_stricto_1* | 0.32 |
| 48 | *Proteobacteria* | *Pasteurellaceae* | *Histophilus* | 0.31 |
| 49 | *Actinobacteria* | *Actinomycetaceae* | *Trueperella* | 0.31 |
| 50 | *Firmicutes* | *Ruminococcaceae* | *Faecalibacterium* | 0.31 |
