# Supplementary material for: High-Fat Diets Led to OTU-Level Shifts in Fecal Samples of Healthy Adult Dogs
Source: Front Microbiol. 2020 Dec 8;11:564160. doi: 10.3389/fmicb.2020.564160 (PMC7752866; doi:10.3389/fmicb.2020.564160)
Supplement: Supplementary file 4 [file Table_4.DOCX]

| **Supplementary Table S4:** Permutation analysis of variance (PERMANOVA) results for both rarefied and unrarefied comparisons of dog fecal microbial communities by treatment. 999 permutations | | | | | | | |
| --- | --- | --- | --- | --- | --- | --- | --- |
|  |  | DF^1^ | SumSq^2^ | MeanSq^3^ | F^4^ | R2^5^ | P-value |
| PERMANOVA  Rarefied  (20,900 seqs) | Treatment | 3 | 0.648 | 0.216 | 0.843 | 0.083 | 0.735 |
|  | Residual | 28 | 7.17 | 0.256 |  | 0.917 |  |
|  | Total | 31 | 7.821 |  |  | 1 |  |
| PERMANOVA  Non-Rarefied | Treatment | 3 | 0.655 | 0.218 | 0.809 | 0.08 | 0.834 |
|  | Residual | 28 | 7.562 | 0.27 |  | 0.92 |  |
|  | Total | 31 | 8.217 |  |  | 1 |  |
| ^1^DF: Degrees of freedom  ^2^SumSq: sum of squares  ^3^MeanSq: mean squares  ^4^F: F-statistic  ^5^R2: coefficient of determination “R-squared” | | | | | | | |
