# Supplementary material for: High-Fat Diets Led to OTU-Level Shifts in Fecal Samples of Healthy Adult Dogs
Source: Front Microbiol. 2020 Dec 8;11:564160. doi: 10.3389/fmicb.2020.564160 (PMC7752866; doi:10.3389/fmicb.2020.564160)
Supplement: Supplementary file 5 [file Table_5.DOCX]

| **Supplementary Table S5:** Functional potential of OTUs with a significant linear increase or decrease in response to an increase in dietary fat | | | | |
| --- | --- | --- | --- | --- |
| **OTU** | **Direction of Change** | **Taxonomic classification**  **(Based on Silva reference)** | **Previously published functional potential** | **Sources** |
| OTU2 | Decreased | *Prevotellaceae_Ga6A1_group* |  |  |
| OTU3 | Decreased | *Fusobacterium* | Utilize amino acids and produce butyrate | Barcenilla et al., 2000 Butowski et al., 2019 |
| OTU9 | Decreased | *Catenibacterium* | Utilizes glucose to produce acetic, lactic, butyric, and iso-butyric acids | Kageyama and Benno, 2000 |
| OTU12 | Increased | *Allobaculum* | Contributes to mucus formation on gut lining | Everard 2014 |
| OTU17 | Decreased | *Alloprevotella* | Saccharolytic bacteria | Qu et al., 2017 |
| OTU21 | Decreased | *Alloprevotella* | Saccharolytic bacteria | Qu et al., 2017 |
| OTU31 | Increased | *Parasutterella* | Bile acid maintenance and cholesterol metabolism | Ju et al., 2019 |
| OTU32 | Increased | *Allobaculum* | Contributes to mucus formation on gut lining | Everard 2014 |
| OTU35 | Increased | *Paeniclostridium* |  |  |
| OTU37 | Decreased | *Bacteroides* | Fermentation of indigestible carbohydrates | Handl et al., 2013 |
| OTU41 | Decreased | *Anaerobiospirillum* | Fermentation of carbohydrates producing succinic and acetic acids | Davis et al., 1979 |
| OTU43 | Decreased | *Romboutsia* | Utilization glucose to produce hydrogen | Gerritsen et al., 2014 |
| OTU44 | Decreased | *Blautia* | Utilization of carbohydrates to produce acetic acid, lactic acid, and ethanol | Liu et al., 2008 |
| OTU45 | Decreased | *Bacteroides* | Fermentation of indigestible carbohydrates | Handl et al., 2013 |
| OTU48 | Increased | *Histophilus* |  |  |
| OTU58 | Decreased | *Allobaculum* | Contributes to mucus formation on gut lining | Everard 2014 |
| OTU62 | Increased | *Succinivibrionaceae_UCG-001* |  |  |
| OTU66 | Decreased | *Lachnoclostridium* | Production of secondary bile acids through bile acid dihydroxylation activity | Ridlon et al., 2015 |
| OTU86 | Decreased | *Lachnospiraceae_ge* |  |  |
| OTU90 | Increased | *Bifidobacterium* | Reduces intestinal endotoxins and improves mucosal barrier function; BSH activity | Wang et al., 2006; Griffiths et al., 2004; Ridlon et al., 2006 |
| OTU96 | Decreased | *Alloprevotella* | Saccharolytic bacteria | Qu et al., 2017 |
| OTU99 | Increased | *Lactobacillus* | Produces lactic acid by metabolizing carbohydrates; BSH activity; modulates the immune system and protects the gut epithelial barrier | Walter 2008; Ridlon et al., 2006; Lebeer et al., 2008 |
